# A comprehensive pancancer analysis reveals the potential value of RAR-related orphan receptor C (RORC) for cancer immunotherapy

**DOI:** 10.3389/fgene.2022.969476

**Published:** 2022-09-15

**Authors:** Shengfu He, Jiawen Yu, Weijie Sun, Yating Sun, Mingyang Tang, Bao Meng, Yanyan Liu, Jiabin Li

**Affiliations:** ^1^ Department of Infectious Diseases, The First Affiliated Hospital of Anhui Medical University, Hefei, China; ^2^ Department of Oncology, Anqing First People’s Hospital of Anhui Medical University/Anqing First People’s Hospital of Anhui Province, Anqing, China; ^3^ Institute of Bacterial Resistance, Anhui Medical University, Hefei, China; ^4^ Anhui Center for Surveillance of Bacterial Resistance, Hefei, China

**Keywords:** RAR-related orphan receptor C, pan-cancer, prognosis, immune microenvironment, immunotherapy

## Abstract

**Background:** RAR-related orphan receptor C (RORC) plays an important role in autoimmune responses and inflammation. However, its function in cancer immunity is still unclear. Its potential value in cancer immunotherapy (CIT) needs to be further studied.

**Methods:** Expression and clinical data for 33 cancers were obtained from UCSC-Xena. The correlation between RORC expression and clinical parameters was analyzed using the limma software package to assess the prognostic value of RORC. Timer2.0 and DriverDBv3 were used to analyze the RORC mutation and methylation profiles. RORC-associated signaling pathways were identified by GSEA. The correlations of RORC expression with tumor microenvironment factors were further assessed, including immune cell infiltration (obtained by CIBERSORT) and immunomodulators (in pancancer datasets from the Tumor-Immune System Interactions and Drug Bank [TISIDB] database). In addition, the correlations of RORC with four CIT biomarkers (tumor mutational burden, microsatellite instability, programmed death ligand-1, and mismatch repair) were explored. Furthermore, three CIT cohorts (GSE67501, GSE168204, and IMvigor210) from the Gene Expression Omnibus database and a previously published study were used to determine the association between RORC expression and CIT response.

**Results:** RORC was differentially expressed in many tumor tissues relative to normal tissues (20/33). In a small number of cancers, RORC expression was correlated with age (7/33), sex (4/33), and tumor stage (9/33). Furthermore, RORC expression showed prognostic value in many cancers, especially in kidney renal clear cell carcinoma (KIRC), brain lower grade glioma (LGG), and mesothelioma (MESO). The mutation rate of RORC in most cancer types was low, while RORC was hypermethylated or hypomethylated in multiple cancers. RORC was associated with a variety of biological processes and signal transduction pathways in various cancers. Furthermore, RORC was strongly correlated with immune cell infiltration, immunomodulators, and CIT biomarkers. However, no significant association was found between RORC and CIT response in the three CIT cohorts.

**Conclusion** Our findings revealed the potential immunotherapeutic value of RORC for various cancers and provides preliminary evidence for the application of RORC in CIT.

## Introduction

Every year, approximately 20 million new cases of cancer are diagnosed and 10 million cancer-related deaths occur around the world (Sung et al., 2021). The occurrence of cancer is closely related to autoimmunity and its progression reflects the inability of the immune system to control the growth of tumor cells. Over the past decades, cancer immunotherapy (CIT) has become an effective clinical strategy. The principle of CIT is to induce the immune system to eliminate tumors, but there are still many patients who are unresponsive or poorly responsive to CIT (Riley et al., 2019). Further investigation into the association of immunity-related factors with cancer will provide reference data for future studies.

To obtain a more suitable microenvironment for growth, tumor cells alter the normal immune microenvironment by secreting immunosuppressive factors and further regulating immune cells, among which CD4^+^ T cells play an important role (Lowery et al., 2022). CD4^+^ T cells can differentiate into various subsets such as T helper cells (Th1, Th2, Th9, Th17, and Th22), regulatory T cells (Tregs), and follicular helper T cells (Saravia et al., 2019). Recently, several studies have confirmed that Th17 and Treg play important roles in cancer and autoimmunity (Knochelmann et al., 2018).

RAR-related orphan receptor C (RORC) is a 24-kb protein-encoding gene located on chromosome 1 (1q21.3). RORγ T, a RORC-encoded protein, is the key transcription factor responsible for Th17 polarization and function, as well as thymocyte development (Yahia-Cherbal et al., 2019). Studies have shown that RORC plays an important role in autoimmunity and inflammation (IvanovMcKenzie et al., 2006; Michelini et al., 2021). Several studies have shown that RORC performs critical regulatory functions in cell proliferation, metastasis, and chemoresistance in various malignancies such as hematologic tumors (Subramanian et al., 2019), breast cancer (Oh et al., 2014), bladder cancer (Cao et al., 2019), and melanoma (Brożyna et al., 2016). The exact role that RORC plays in other cancers is still unknown.

In this study, RORC expression was evaluated in 33 cancer types and the effects of RORC on the tumor immune microenvironment were determined. Furthermore, the associations of RORC expression with many immunomodulators and dynamic immunological biomarkers were investigated. In brief, this study investigated the role of RORC in the pancancer immune microenvironment and explored the potential value of RORC in CIT, which may be helpful for future studies.

## Materials and methods

### Data collection

RNA-seq data on 33 human cancer types were obtained from The Cancer Genome Atlas (TCGA) database (https://portal.gdc.cancer.gov/). Abbreviations and full names of the 33 cancers are shown in Table 1. RORC expression data in normal tissues was downloaded from the Genotype-Tissue Expression (GTEx) database (https://commonfund.nih.gov/GTEx/). Corresponding clinical and prognostic data were acquired from the University of California Santa Cruz (UCSC) Xena Explorer. Additionally, RNA-seq and clinical data from the following three CIT cohorts were downloaded from the Gene Expression Omnibus (GEO) database (https://www.ncbi.nlm.nih.gov/geo/) and a previously published study (Mariathasan et al., 2018): 1) IMvigor 210 (34 patients with bladder cancer treated with the programmed death ligand 1 [PD-L1] inhibitor atezolizumab) (Mariathasan et al., 2018), 2) GSE67501 (patients with renal cell carcinoma treated with programmed cell death protein 1 [PD-1] inhibitor), and 3) GSE168204 (patients with metastatic melanoma treated with PD-1 inhibitor).

**TABLE 1:** Full names of 33 cancer types and the numbers of tumor and adjacent normal tissues from TCGA database.

Abbreviation	Full name	Tumor tissue	Adjacent normal tissue
ACC	Adrenocortical carcinoma	79	0
BLCA	Bladder urothelial carcinoma	408	19
BRCA	Breast invasive carcinoma	1098	113
CESC	Cervical squamous cell carcinoma and endocervical	306	3
CHOL	Cholangiocarcinoma	36	9
COAD	Colon adenocarcinoma	458	41
DLBC	Lymphoid neoplasm diffuse large B-cell lymphoma	48	0
ESCA	Esophageal carcinoma	162	11
GBM	Glioblastoma multiforme	167	5
HNSC	Head and neck squamous cell carcinoma	502	44
KICH	Kidney chromophobe	65	24
KIRC	Kidney renal clear cell carcinoma	531	72
KIRP	Kidney renal papillary cell carcinoma	289	32
LAML	Acute myeloid leukemia	151	0
LGG	Brain lower grade glioma	525	0
LIHC	Liver hepatocellular carcinoma	373	50
LUAD	Lung adenocarcinoma	515	59
LUSC	Lung squamous cell carcinoma	501	49
MESO	Mesothelioma	86	0
OV	Ovarian serous cystadenocarcinoma	379	0
PAAD	Pancreatic adenocarcinoma	178	4
PCPG	Pheochromocytoma and paraganglioma	183	3
PRAD	Prostate adenocarcinoma	496	52
READ	Rectum adenocarcinoma	167	10
SARC	Sarcoma	263	2
SKCM	Skin cutaneous melanoma	471	1
STAD	Stomach adenocarcinoma	375	32
TGCT	Testicular germ cell tumors	156	0
THCA	Thyroid carcinoma	510	58
THYM	Thymoma	119	2
UCEC	Uterine corpus endometrial carcinoma	544	35
UCS	Uterine carcinosarcoma	56	0
UVM	Uveal melanoma	80	0

### RAR-related orphan receptor C expression in 33 cancer types

Differences in RORC expression between tumor and normal tissues for 33 cancer types were analyzed using the limma package in R Studio software. To further analyze the differences in RORC expression in tumor tissues and normal tissues, we combined the TCGA data with GTEx data. The numbers of “healthy tissue” samples from the GTEx database are presented in Table 2. The Wilcoxon test was also used to analyze differences in RORC expression stratified by various clinical characteristics (age, sex, and tumor stage).

**TABLE 2:** Number of healthy tissues from GTEx database.

Tissue	Number	Tissue	Number	Tissue	Number
Adipose Tissue	515	Adrenal Gland	128	Bladder	9
Blood	444	Blood Vessel	606	Bone Marrow	70
Brain	1152	Breast	179	Cervix Uteri	10
Colon	308	Esophagus	653	Fallopian Tube	5
Heart	377	Kidney	28	Liver	110
Lung	288	Muscle	396	Nerve	278
Ovary	88	Pancreas	167	Pituitary	107
Prostate	100	Salivary Gland	55	Skin	812
Small Intestine	92	Spleen	100	Stomach	174
Testis	165	Thyroid	279	Uterus	78
Vagina	85				

### Prognostic value of RAR-related orphan receptor C expression

Cox regression analysis was performed to investigate the prognostic value of RORC in 33 cancers, based on overall survival (OS), disease-free survival (DFS), disease-specific survival (DSS), and progression-free survival (PFS). Hazard ratio (HR) > 1 represents a risk factor for death. *p* < 0.05 was considered significant. Kaplan–Meier survival curve analyses were also conducted for multiple cancer types. *p* < 0.05 was considered significant.

### RAR-related orphan receptor C mutation and methylation profiles

TIMER2.0 (http://timer.comp-genomics.org/) was used to create a bar plot showing the RORC mutation rate for each TCGA cancer type (Li et al., 2020). DriverDBv3 (http://driverdb.tms.cmu.edu.tw/) is a cancer omics database that incorporates somatic mutation, methylation, copy number variation, RNA expression, miRNA expression and clinical data in addition to annotation bases (Liu et al., 2020). We explored the RORC mutation rates using both TIMER2.0 and DriverDBv3. Moreover, the RORC methylation types in multiple cancer types were obtained from DriverDBv3.

### Gene set enrichment analysis (GSEA)

GSEA is commonly used to analyze differences in the levels of biological processes and pathways between two biological states in transcriptomics research (Powers et al., 2018). The gene set files c5. go.v7.4. symbols.gmt and c2. cp.kegg.v7.4. symbols were obtained from GSEA software (https://www.gsea-msigdb.org/gsea/index.jsp). GSEA was then used to analyze the biological processes and signaling pathways associated with RORC in 33 cancer types.

### Correlations of RAR-related orphan receptor C expression with immune-related factors

Estimation of STromal and Immune cells in MAlignant Tumors using Expression data (ESTIMATE) is an algorithm that uses gene expression signatures to infer the proportion of stromal and immune cells in tumor samples (Yoshihara et al., 2013). The immune score and stromal score were calculated by ESTIMATE. Spearman’s correlation coefficient was used to analyze the correlations of RORC expression with the immune and stromal scores.

Cell-type Identification By Estimating Relative Subsets Of RNA Transcripts (CIBERSORT) is a method for characterizing the cell composition of complex tissues based on gene expression profiles (Chen et al., 2018). We used the CIBERSORT algorithm to determine the infiltration of 22 lymphocyte subsets in the samples. Spearman’s correlation coefficient was used to analyze the correlations of RORC expression with the infiltration of the 22 lymphocyte subsets.

Tumor-Immune System Interactions and Drug Bank (TISIDB; http://cis.hku.hk/TISIDB/index.php) is a web portal dedicated to collecting data on the interactions between tumors and the immune system, and it integrates multiple heterogeneous datasets (Ru et al., 2019). The correlations of RORC expression with immunomodulators (immunoinhibitors, immunostimulators, and major histocompatibility complex (MHC) molecules) were investigated using TISIDB.

Additionally, PD-L1, tumor mutational burden (TMB), microsatellite instability (MSI), and mismatch repair (MMR) are crucial biomarkers that predict the CIT response (Rizzo et al., 2021). Therefore, the correlations of RORC expression with PD-L1, TMB, MSI, and MMR were further investigated using Spearman’s correlation coefficient. TMB was determined using the PERL programming language (version 5.32.1). MSI data were obtained from a previously published study (Bonneville et al., 2017).

### Correlations of RAR-related orphan receptor C expression with CIT responses

Data on RORC expression and CIT responses [responsive, which was defined as complete response (CR) or partial response (PR), and non-responsive, which was defined as progressive disease (PD) or stable disease (SD)] were obtained from three relevant independent CIT cohorts (IMvigor210, GSE67501, and GSE168204). The Wilcoxon test was then used to analyze the differences in CIT response between the high and low RORC expression groups, and *p* < 0.05 was used to indicate a significant difference.

### Statistical analysis

All statistical analyses were performed using R software (version 4.1.0). Correlation analyses were performed using Spearman’s correlation coefficient. Analyses of differential RORC gene expression were performed using Wilcoxon tests. *p* < 0.05 was considered to indicate statistical significance.

## Results

### RAR-related orphan receptor C expression in 33 cancers

We explored the differences in RORC expression between tumor and normal tissues in 33 human cancers. RORC was differentially expressed in 12 cancers compared to adjacent normal tissues (BRCA, CHOL, COAD, ESCA, HNSC, KIRP, LUAD, LUSC, PRAD, READ, STAD, and THCA), with RORC expression being significantly higher in BRCA, LUAD, and PRAD than in adjacent normal tissues (Figure 2A). Figures 2B,C show RORC expression in various cancers and various normal tissues (GTEx data), respectively. Figure 2D shows the results of the combined analysis of TCGA and GTEx data, indicating that RORC expression was higher in BRCA, COAD, LUAD, OV, UCEC, and UCS than in normal tissues, but lower in ACC, CHOL, HNSC, KIRP, LAML, LGG, LIHC, LUSC, PAAD, READ, SKCM, STAD, TGCT, and THCA.

**FIGURE 1:**
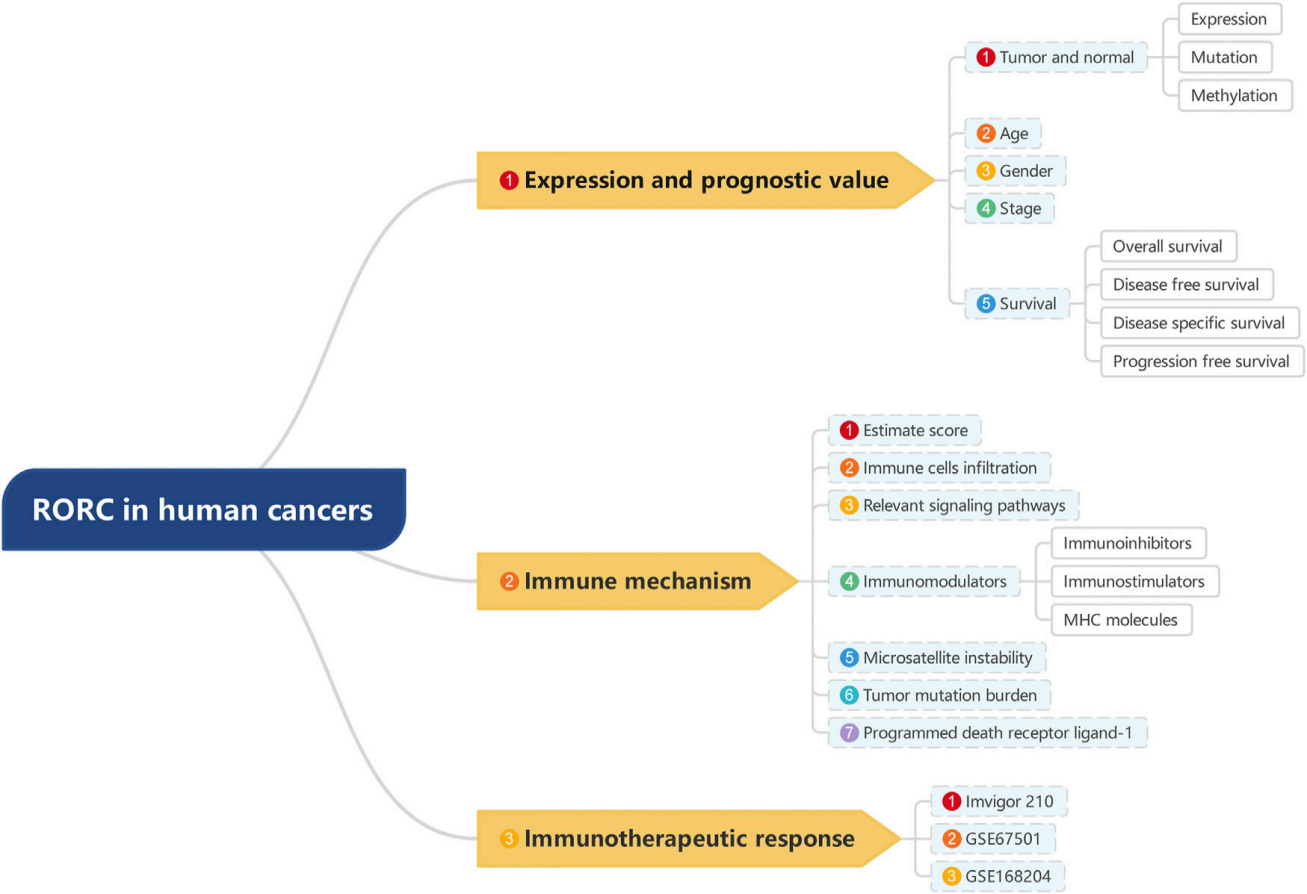
Analyses and indicators employed in our research.

**FIGURE 2:**
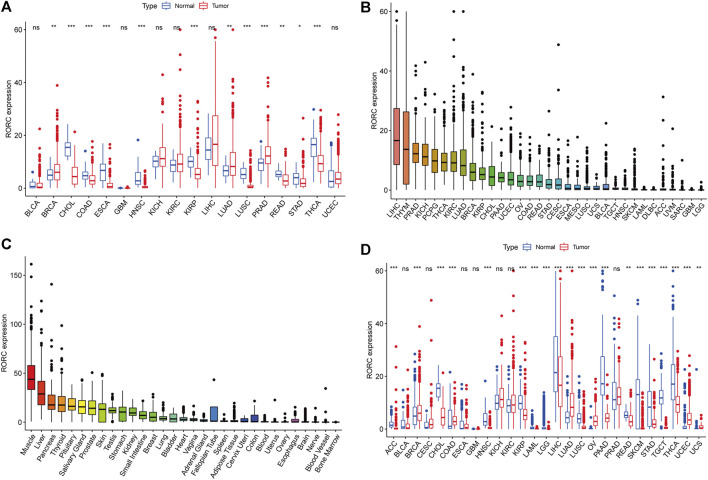
Differential RORC expression between cancer and normal tissues. **(A)** Differential RORC expression between cancer and adjacent normal tissues based on TCGA data (cancers with <5 adjacent normal tissue samples were discarded). **(B)** Mean RORC expression in 33 cancer tissues (from high to low). **(C)** Mean RORC expression in normal tissues based on GTEx data (from high to low). **(D)** Differential RORC expression between cancer and normal tissues in various cancers based on combined TCGA and GTEx data.

**FIGURE 3:**
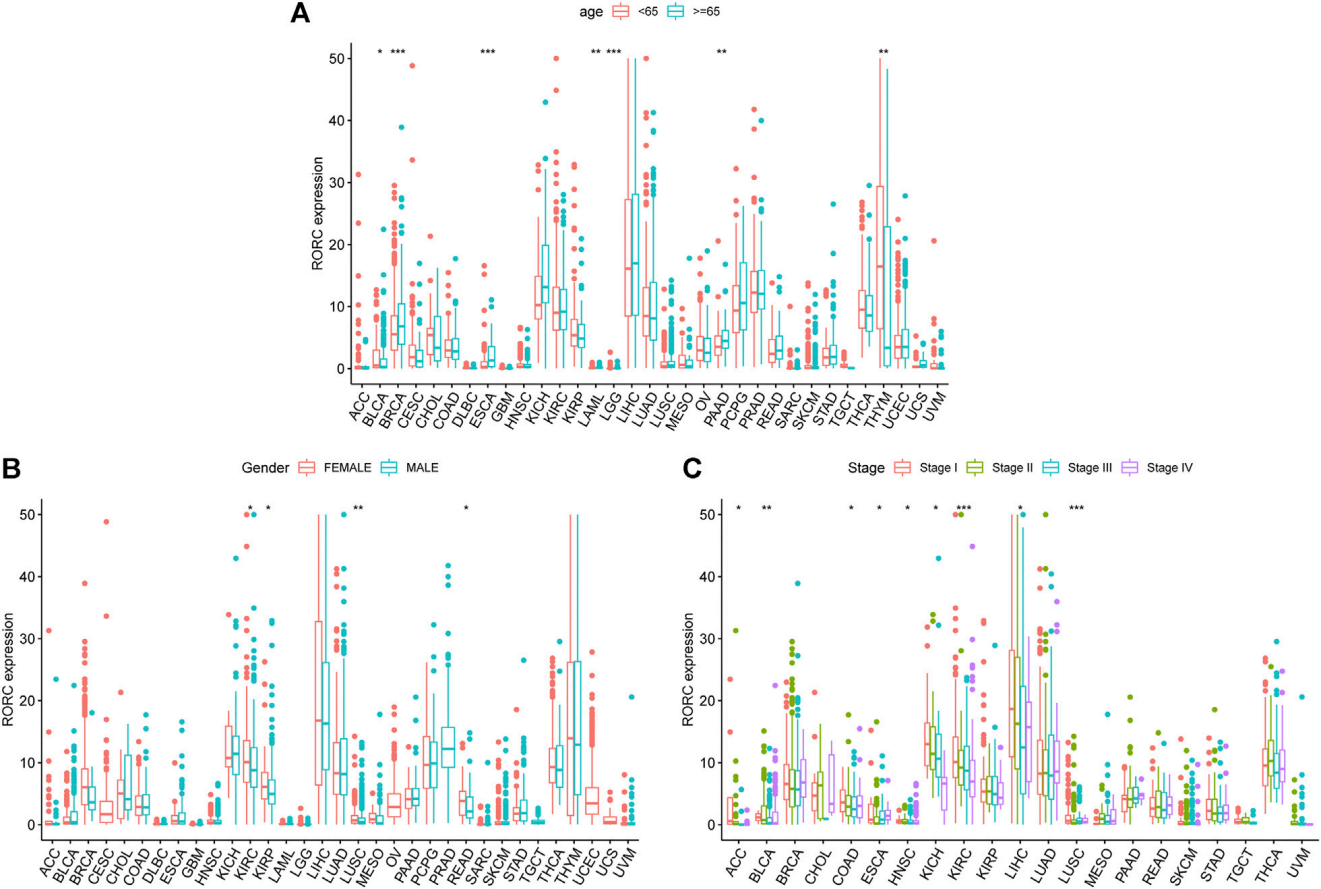
Associations of RORC with **(A)** age, **(B)** sex, and **(C)** tumor stage. *p < 0.05, **p < 0.01, and ***p < 0.001.

We also explored the associations between RORC expression and clinical features in 33 human cancers. RORC expression significantly differed by age (<65 vs. ≥ 65 years) in BLCA, BRCA, ESCA, LAML, LGG, PAAD, and THYM (Figure 3A), sex (higher in females) in KIRC, KIRP, LUSC, and READ (Figure 3B), and tumor stage in ACC, BLCA, COAD, ESCA, HNSC, KICH, KIRC, LIHC, and LUSC (Figure 3C).

### RAR-related orphan receptor C mutation and methylation profiles

**FIGURE 4:**
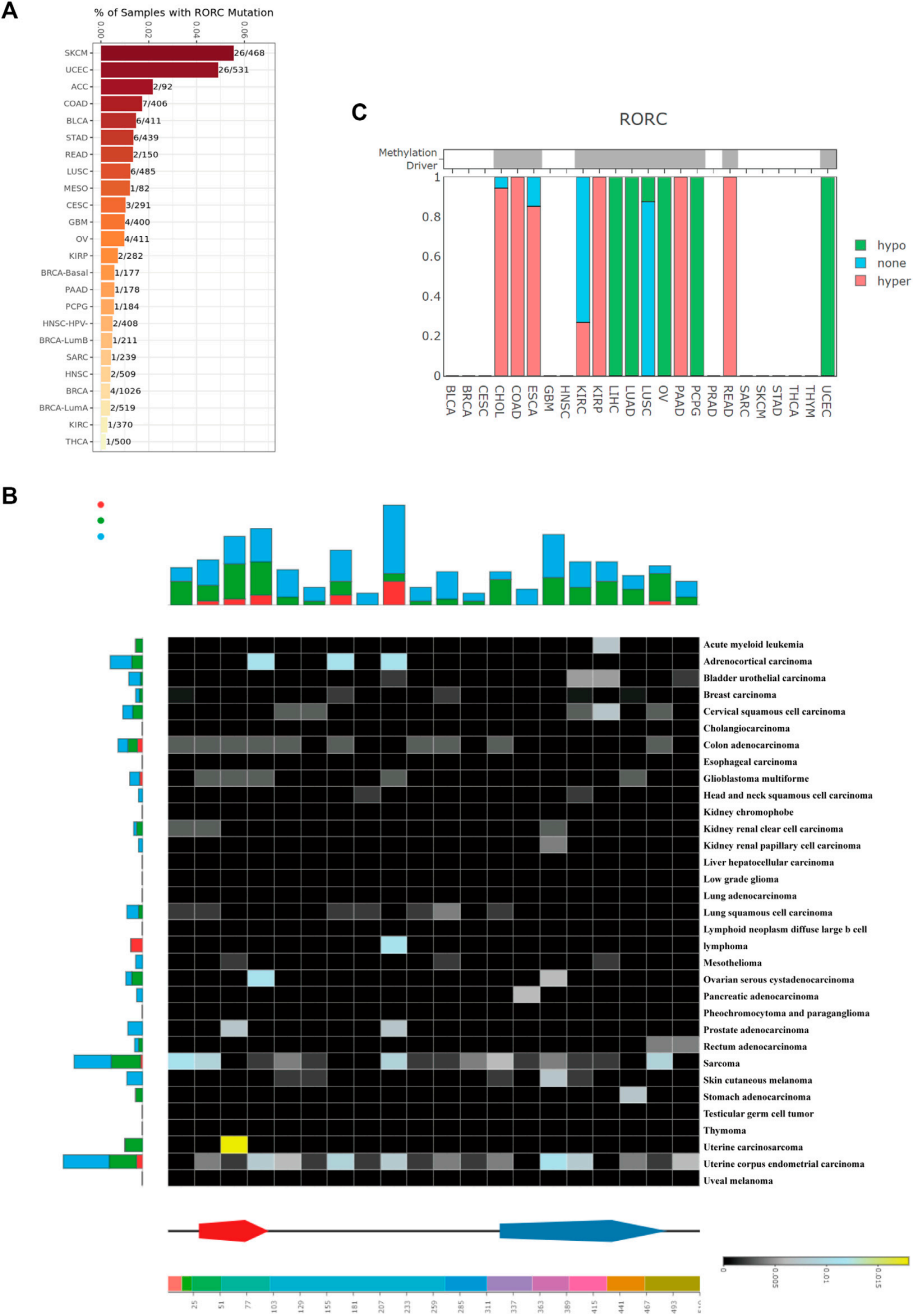
RORC mutation and methylation analysis in various cancers. RORC mutation analysis using **(A)** TIMER 2.0, and **(B)** DriverDBv3 data. **(C)** RORC methylation type in various cancers.

To explore the mechanisms underlying differential RORC expression in various cancers, we explored the RORC mutation rate in multiple cancer types in the TCGA database using TIMER2.0 (Figure 4A) and DriverDBv3 (Figure 4B). TIMER2.0 showed that RORC had the highest mutation rate in SKCM (26/468) and the lowest in THCA (1/500). DriverDBv3 showed that the RORC mutation rate in most cancers was low. Figure 4C shows the methylation type of RORC in multiple cancer types. Interestingly, RORC was hypermethylated in CHOL, COAD, ESCA, KIRP, PAAD, and READ, but hypomethylated in LIHC, LUAD, LUSC, OV, PCPG, and UCEC.

### Prognostic value of RAR-related orphan receptor C expression

**FIGURE 5:**
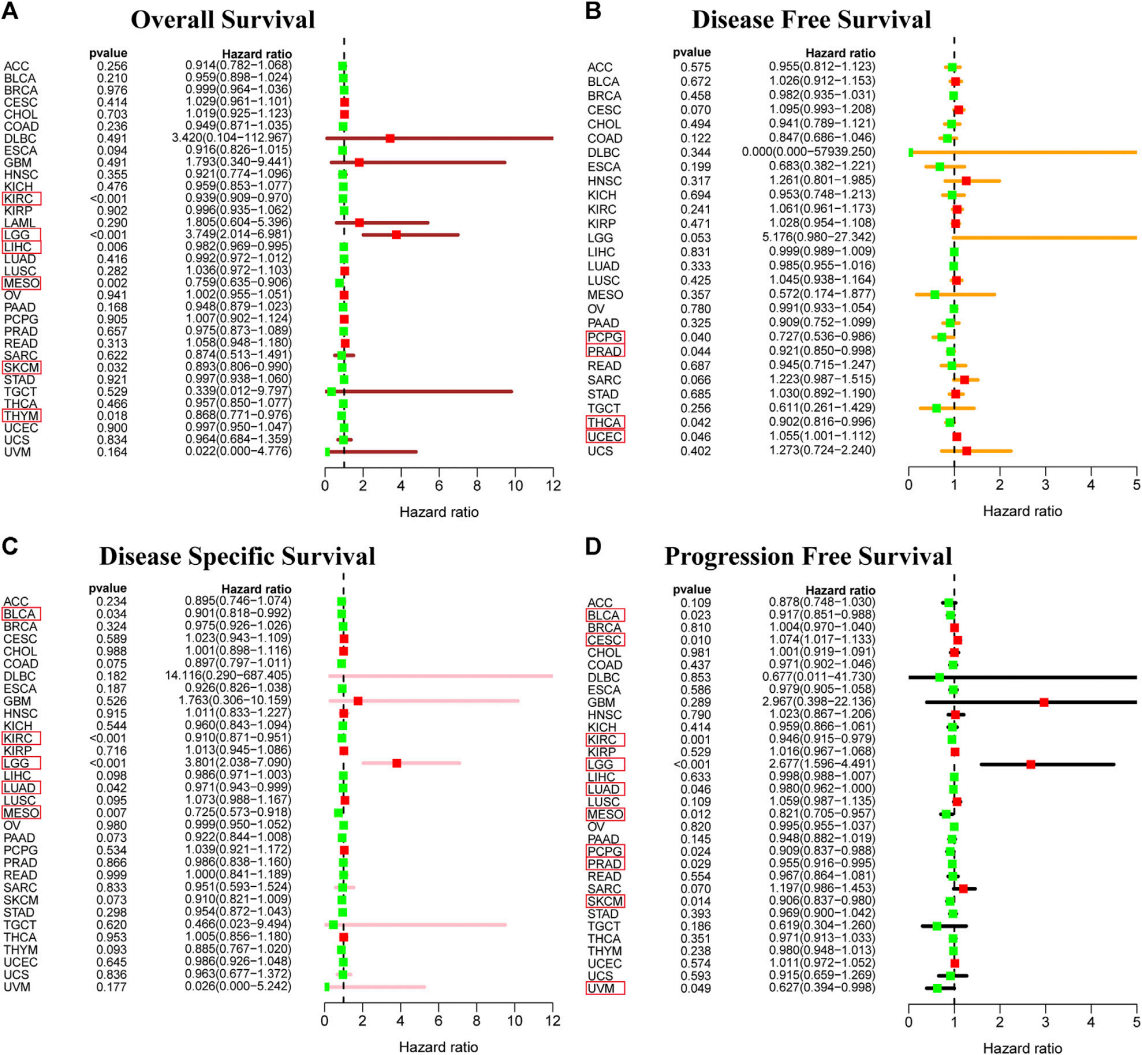
Prognostic value of RORC. Correlations of RORC expression with **(A)** OS, **(B)** DFS, **(C)** DSS, and **(D)** PFS. RORC expression was significantly correlated with the prognosis of the cancers in red box (*p <* 0.05). Hazard ratio >1 indicates that RORC expression was a risk factor for death.

To determine the correlation of RORC expression with prognosis (OS, DFS, DSS, and PFS) in 33 tumors, we used the survival and survminer R packages. RORC expression was a risk factor for OS in LGG patients, but a protective factor in KIRC, LIHC, MESO, SKCM, and THYM (Figure 5A). It was a risk factor for DFS in UCEC but a protective factor in PCPG, PRAD, and THCA (Figure 5B). It was a risk factor for DSS in LGG but a protective factor in BLCA, KIRC, LUAD and MESO (Figure 5C). It was a risk factor for PFS in LGG and CESC but a protective factor in BLCA, KIRC, LUAD, MESO, PCPG, PRAD, SKCM, and UVM (Figure 5D).

To analyze the relationship between RORC expression and cancer prognosis more comprehensively, Kaplan-Meier survival curve analyses for 33 cancer types were conducted. RORC was significantly associated with OS in ACC, BLCA, GBM, KIRC, LIHC, MESO, THCA, THYM, and UVM (Supplementary Figure 1A), DFS in CESC, HNSC, PAAD, and THCA (Supplementary Figure 1B), DSS in ACC, BLCA, GBM, KIRC, LIHC, LUSC, MESO, PAAD, and THYM (Supplementary Figure 1C), and PFS in ACC, BLCA, CESC, KIRC, LGG, MESO, PAAD, THCA, and UVM (Supplementary Figure 1D).

### Correlations of RAR-related orphan receptor C expression with immune cell infiltration

**FIGURE 6:**
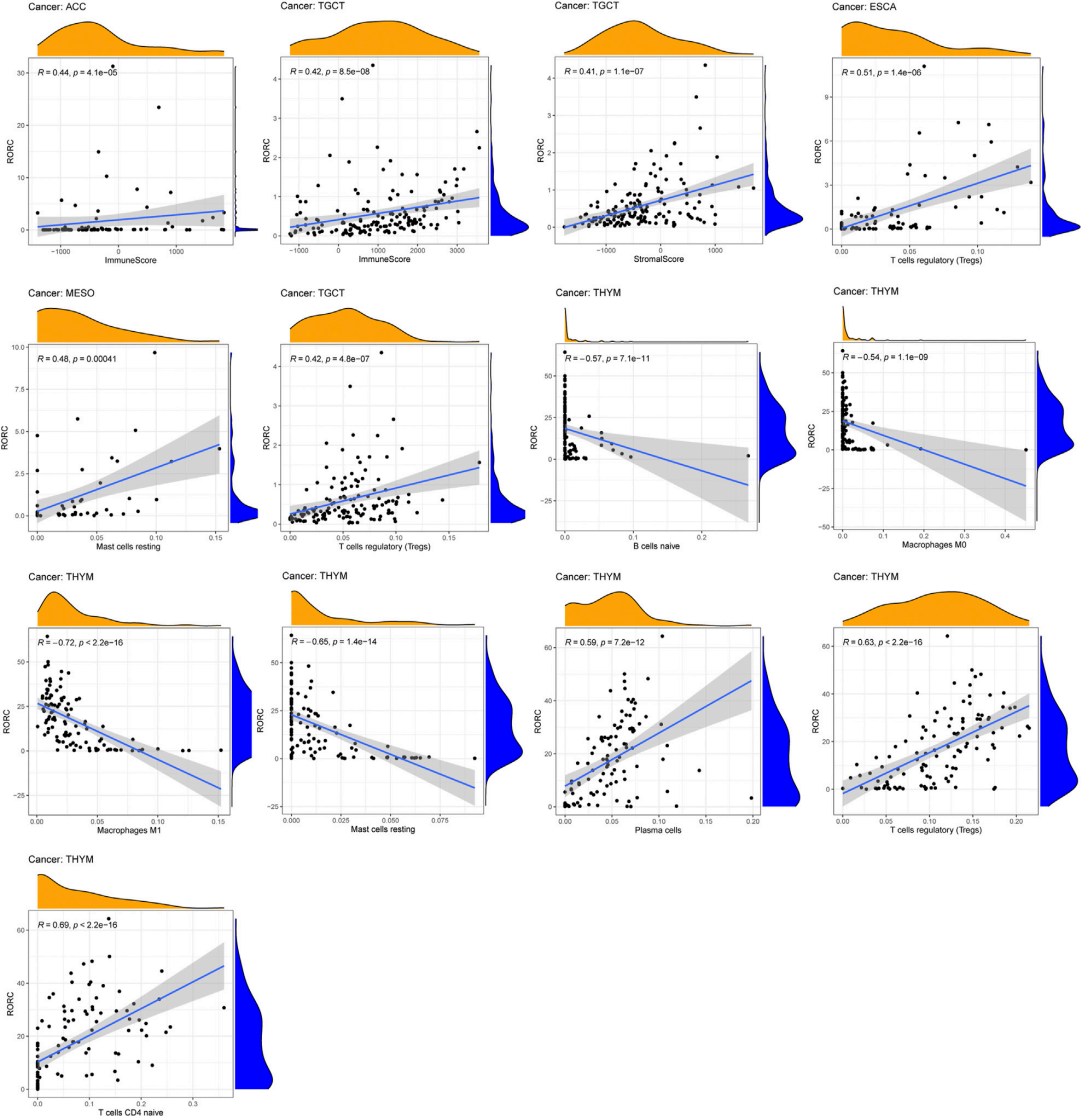
Correlations of RORC expression with ESTIMATE scores and immune cell infiltration. Correlations are shown if R > 0.5 and *p* < 0.05.

Tumor-infiltrating immune cells, as an important part of the tumor microenvironment, are closely related to the occurrence, progression, and metastasis of cancer. We explored the correlation of RORC with the immune and stromal scores, and on immune cell infiltration (thresholds: correlation coefficient >0.4, *p* < 0.001). RORC expression was positively correlated with the immune score in TGCT and ACC and the stromal score in TGCT (Figure 6). RORC expression was positively correlated with Treg infiltration in ESCA and TGCT and mast cell infiltration in MESO. In contrast, it was negatively correlated with mast cell, B lymphocyte, M0 macrophage, and M1 macrophage infiltration in THYM, but positively correlated with plasma cell, Treg, and naive CD4^+^ T cell infiltration.

### Correlations of RAR-related orphan receptor C expression with immunomodulators

**FIGURE 7:**
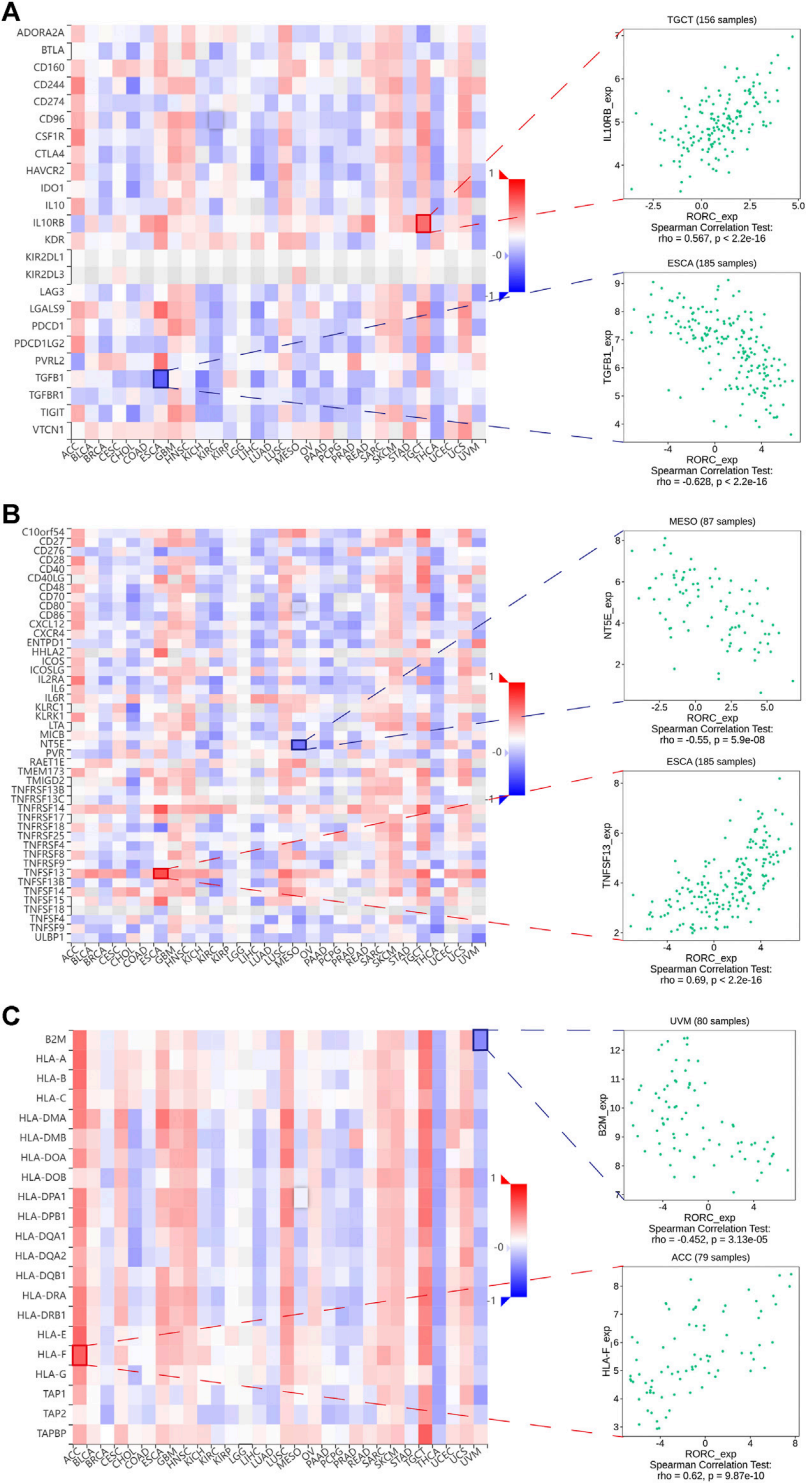
Correlations of RORC expression with **(A)** immunoinhibitors, **(B)** immunostimulators, and **(C)** MHC molecules. Red indicates positive correlation; blue indicates negative correlation. The strongest negative and positive correlations are presented on the right.

To further investigate the potential value of RORC in CIT, we explored the correlations of RORC expression with immunomodulators. Figure 7A demonstrates the correlations between RORC expression and 24 immunoinhibitors in a pancancer analysis. RORC expression exhibited the strongest positive correlation with IL10RB in TGCT, while it exhibited the strongest negative correlation with TGFB1 in ESCA. Figure 7B shows the correlations between RORC and 45 immunostimulators in a pancancer analysis. RORC expression exhibited the strongest positive correlation with TNFSF13 in ESCA, while it exhibited the strongest negative correlation with NT5E in MESO. Figure 7C shows the correlations between RORC expression and 21 MHC molecules in a pancancer analysis. RORC expression exhibited the strongest positive correlation with HLA-F in ACC, while it exhibited the strongest negative correlation with B2M in UVM.

**FIGURE 8:**
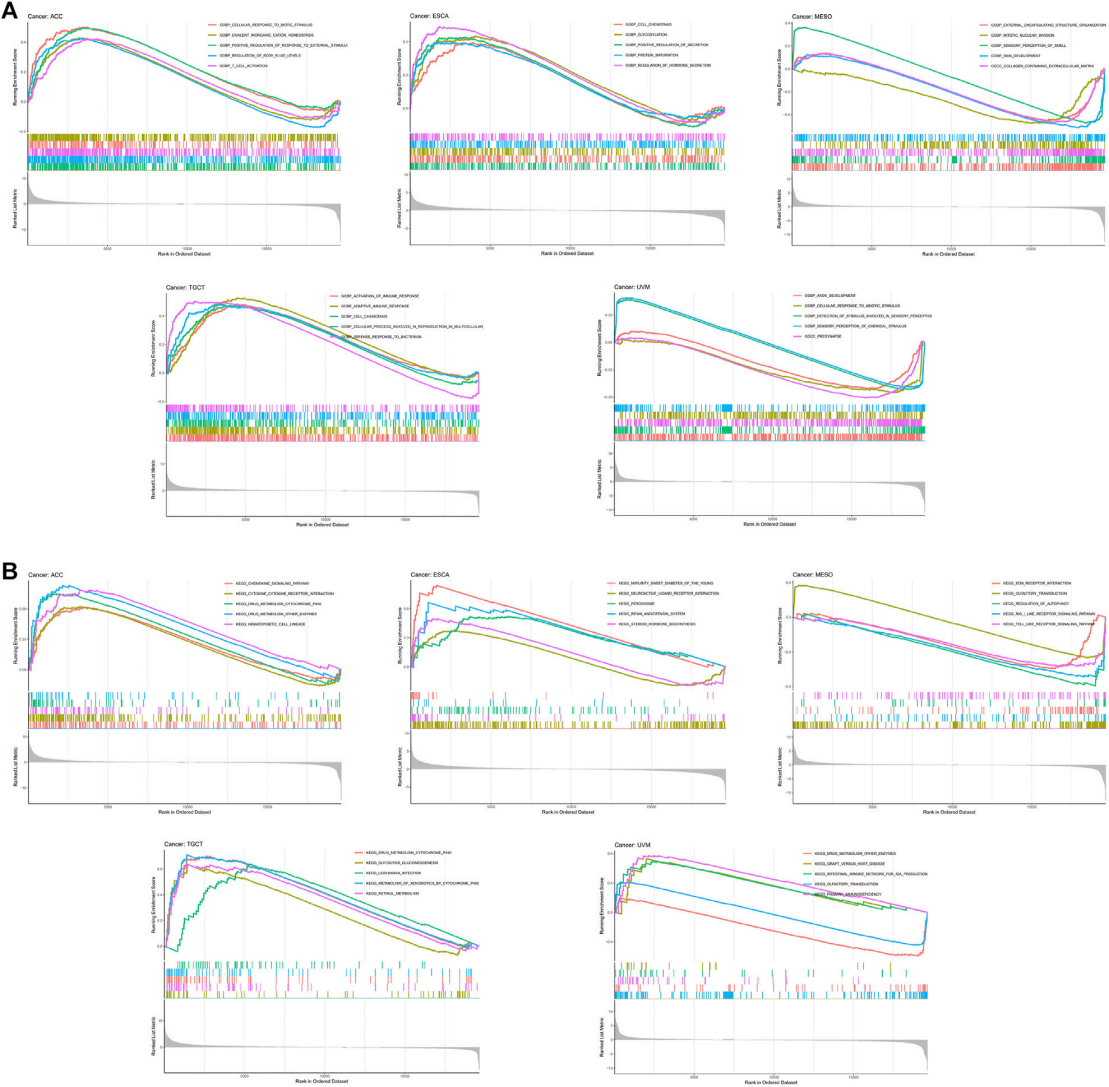
GSEA showing the associations of RORC expression with **(A)** biological processes and **(B)** signaling pathways in five cancers.

Taken together, our results revealed particularly strong correlations between RORC and immunomodulators in ACC, ESCA, MESO, TGCT, and UVM. Interestingly, the biological processes involving RORC (according to GSEA) differed among these five cancers (Figure 8A). In particular, RORC was associated with T cell activation in ACC, and activation of the immune response and the adaptive immune response in TGCT. Additionally, the signaling pathways involving RORC (according to GSEA) were strikingly different (Figure 8B). Remarkably, RORC was associated with the Toll-like receptor (TLR) and Rig-I-like receptor (RLR) signaling pathways in MESO, but was also associated with the intestinal immune network.

### Potential value of RAR-related orphan receptor C in CIT

**FIGURE 9:**
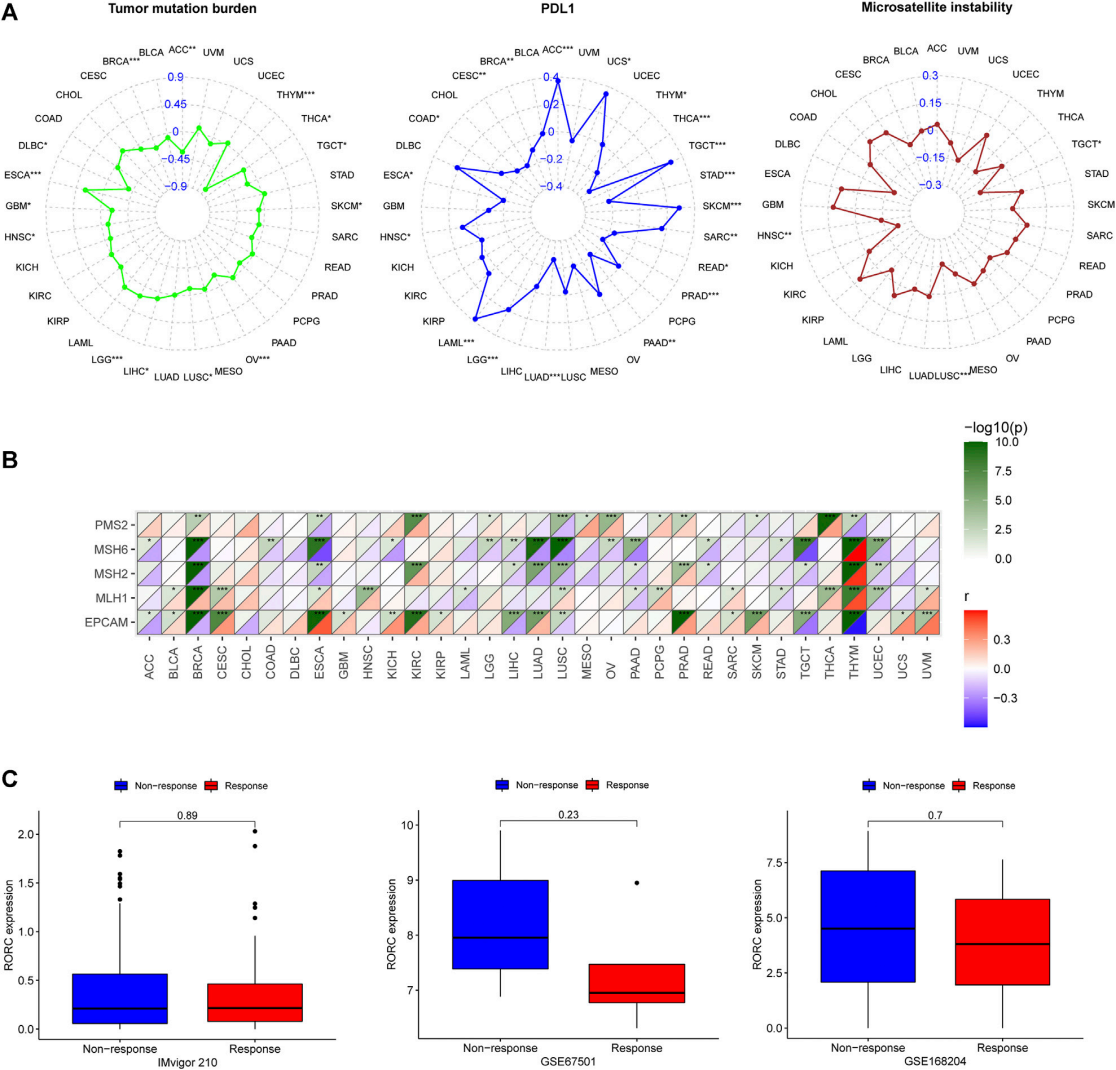
Correlations of RORC expression with **(A)** cancer immunotherapy (CIT) biomarkers (TMB, PD-L1, and MSI) and **(B)** MMR-related genes (PMS2, MSH6, MSH2, MLH1, and EPCAM) and **(C)** differences in RORC expression between responder and non-responder groups in three CIT cohorts. *p < 0.05, **p < 0.01, ***p < 0.001.

TMB, PD-L1, and MSI are effective CIT biomarkers. The correlations between RORC expression and these three CIT biomarkers are shown in Figure 9A. RORC was positively correlated with TMB in LIHC, LGG, and ESCA, but negatively correlated in ACC, THYM, THCA, TGCT, SKCM, OV, LUSC, HNSC, GBM, DLBC, and BRCA. RORC expression was positively correlated with PD-L1 expression in ACC, UCS, TGCT, SKCM, SARC, LGG, LAML, and HNSC, but negatively correlated in THYM, THCA, STAD, READ, PRAD, PAAD, LUAD, ESCA, COAD, CESC, and BRCA. Interestingly, RORC expression was not significantly correlated with MSI in most cancers, but was negatively associated with MSI in TGCT, LUSC, and HNSC. The correlations of RORC expression with mismatch repair (MMR) were also investigated. RORC expression was highly correlated with MMR-related genes (including MLH1, MSH2, MSH6, PMS2, and EPCAM) in BRCA, ESCA, LUSC, and THYM (Figure 9B).

To verify the role of RORC in CIT, we investigated the association of RORC expression with CIT responses in three independent CIT cohorts (IMvigor 210, GSE67501, and GSE168204). In all three cohorts, RORC expression did not differ significantly between the responder and nonresponder groups (Figure 9C). However, there was a non-significant trend indicating that low RORC expression rendered tumors more responsive to CIT.

## Discussion

Cancer has always been a major public health problem worldwide and is the second leading cause of death after cardiovascular diseases (Global Burden of Disease Cancer et al., 2022). Currently, the main cancer treatment methods include surgery, radiotherapy, chemotherapy, and targeted therapy. It is difficult to completely eradicate tumors with conventional surgical treatment, which is always accompanied by a high risk of postoperative recurrence. Radiotherapy and chemotherapy kill normal cells as well as tumor cells and cause many side effects (Islam et al., 2019; Williams et al., 2020). Although targeted drugs may reduce adverse drug reactions, many tumors are prone to develop resistance against these drugs, leading to tumor recurrence (Holohan et al., 2013). With the development of molecular biology and oncobiology, CIT has become a new type of cancer treatment that effectively overcomes certain shortcomings of traditional treatment methods (McNutt, 2013). However, there are various ways in which cancer cells can evade the immune system and become unresponsive or poorly responsive to CIT.

RORγ T, a specific transcription factor encoded by RORC, plays a major regulatory role in Th17 cell polarization and function and is associated with autoimmunity and inflammation (IvanovMcKenzie et al., 2006). Recently, several studies have found that RORC is involved in the regulation of hematological tumors, breast cancer, bladder cancer, and melanoma (Oh et al., 2014; Brożyna et al., 2016; Cao et al., 2019; Subramanian et al., 2019). However, the exact role of RORC in other cancers remains unknown, although comprehensive studies of differences in RORC expression between tumor and normal tissues have revealed its potential value in CIT for a variety of cancer types.

Our study showed that there were significant differences in RORC expression between tumor and normal tissues (adjacent normal tissues [TCGA data] and healthy tissues [GTEx data]) in some cancers. Specifically, RORC expression was significantly higher in BRCA, COAD, LUAD, OV, UCEC, and UCS. Similarly, Lin et al. (Lin et al., 2015) found that RORC expression increases in BRCA, while Dong et al. (Dong et al., 2021) found that RORC expression in LUAD was higher than in normal tissues.

It is well known that gene mutation and methylation can alter gene expression (Yang et al., 2014; Jia and Zhao, 2017). Our results showed that the RORC mutation rate in most cancer types was low, whereas RORC methylation significantly differed between cancer and normal tissues in several cancers. Interestingly, abnormal RORC methylation occurred in the majority of cancers with differential RORC expression, which indicates that differential RORC expression may be caused by abnormal methylation in some cancers.

We found that RORC expression was higher in older patients (≥65 years) than younger patients (<65 years) in LAML. Notably, Subramanian et al. (Subramanian et al., 2019) found that RORC expression decreased with age in human T lymphocytic virus (HTLV)-1-infected patients who did not develop adult T-cell leukemia (which can occur decades after HTLV-1 infection (Nakahata et al., 2011)), demonstrating that an age-dependent decline in RORC expression indicates a possible early event in HTLV-1-driven leukemogenesis. We also found that RORC expression was not sex-specific in most cancers, and RORC expression varied with tumor stage in some cancers. Similarly, a previous study reported that RORC expression in BLCA decreases with increasing tumor stage (Cao et al., 2019). Notably, RORC expression had prognostic value in some cancers, especially KIRC, LGG, and MESO. Consistently, Ait Ssi et al. (Ait Ssi et al., 2021) found that RORC expression is upregulated in LGG patients with a poor prognosis. In conclusion, RORC expression can be an important regulator of cancers and RORC has prognostic value in some cancers.

The immune microenvironment is a vital feature of tumors; it can be used to predict the clinical prognosis of patients and it also plays an important role in predicting the response to CIT (Hanahan and Weinberg, 2011). Several studies (Paijens et al., 2021; Zheng et al., 2021) have confirmed that tumors are locally infiltrated with various immune cell subsets, including macrophages, dendritic cells, mast cells, natural killer cells, CD4^+^ T cells, and CD8^+^ T cells. Different types of tumors have different levels of immune cell infiltration, and even different patients with the same type of tumor have different levels of immune cell infiltration, which is one reason why patients respond differently to CIT (Wu and Dai, 2017).

The results of our study showed that RORC expression was positively correlated with immune and stromal scores as well as Tregs. Tregs play a crucial role in maintaining immune tolerance and repressing antitumor immunity (Knochelmann et al., 2018). Siska et al. (Siska et al., 2017) showed there was higher Treg infiltration in advanced TGCT. Moreover, the authors suggested that an increase in Treg infiltration may lead to a poor CIT response in nonspermatogonial germ cell tumors. Interestingly, RORC expression was positively correlated with Tregs, naive CD4^+^ T cells, and plasma cells in THYM, but was negatively correlated with naive B cells, M0 macrophages, M1 macrophages, and resting mast cells in THYM. In cases of THYM, the imbalance between Th17/Tregs in THYM may be the pathological mechanism underlying the development of myasthenia gravis (MG) (Chen et al., 2021a). Furthermore, the increase in plasma cell levels in MG patients has previously been confirmed (Kohler et al., 2013). Moreover, impaired macrophage polarization affects T cell apoptosis and T cell-mediated autoimmunity (Ghosh et al., 2015; Veremeyko et al., 2018). Thus, RORC may contribute to the development of MG by altering the infiltration of immune cells in THYM. Thus, RORC may be a potential target for the treatment of MG in patients with THYM.

We also investigated the biological processes and signaling pathways involving RORC in cancers. TLRs and RLRs play a vital role in cancer immunity (Bourquin et al., 2020). Notably, TLR and RLR signaling pathways were enriched in the high RORC expression subgroup of MESO patients. Similarly, a previous study demonstrated that TLR and Rig-I contribute to the progression of malignant MESO (Wörnle et al., 2009), which means that RORC may be a potential CIT target for use in MESO patients. In short, RORC is an important gene in the immune microenvironment in cancer patients.

Regarding the negative correlations between RORC expression and immunostimulators, RORC expression exhibited the strongest negative correlation with NT5E in MESO. NT5E encodes CD73, which is an ectonucleotidase present on cancer cells that helps cancer cells convert ATP into adenosine (Kordaß et al., 2018). Adenosine is an effective immunomodulator that inhibits antitumor immune responses and promotes metastasis (Allard et al., 2017). Al-Taei et al. (Al-Taei et al., 2017) reported the regulatory function of CD73 in the immune microenvironment in MESO, demonstrating the promising role of RORC in CIT for the treatment of MESO. Regarding the positive correlations between RORC expression and immunostimulators, RORC expression exhibited the strongest positive correlation with TNFSF13 in ESCA. TNFSF13 is a proliferation-inducing ligand of the tumor necrosis factor (TNF) superfamily. It plays an important role in autoimmunity and has also been reported as a potential molecular target to overcome immunosuppression (Chen et al., 2021b). Studies have shown that high TNFSF13 expression in tumor cells and fibroblasts is associated with a poor prognosis of non-small cell lung cancer, and TNFSF13 also promotes the development of LAML (Qian et al., 2014; Chapellier et al., 2019). Furthermore, TNFSF13 plays a crucial role in triple-negative breast cancer, laryngeal squamous cell carcinoma, gastric cancer, and glioma (Wang et al., 2016; Lin et al., 2020; Chen et al., 2021b; Zhang et al., 2021).

Regarding the negative correlations between RORC expression and immunoinhibitors, RORC expression exhibited the strongest negative correlation with TGFB1 in ESCA. Recently, Talukdar et al. demonstrated that TGFB1 expression influences the prognosis of ESCA (Talukdar et al., 2021). Furthermore, several studies have reported that high TGFB1 expression is associated with poor prognosis in many cancers (Liang et al., 2020; de Streel and Lucas, 2021). These findings further confirmed that RORC may be a potential CIT target for treating ESCA. Regarding the positive correlations between RORC expression and immunoinhibitors, RORC expression exhibited the strongest positive correlation with IL10RB in TGCT. As Hanna et al. (Hanna et al., 2021) reported, IL10RB can promote antitumor immunity by maintaining the PD-1^int^TCF-1^+^CD8^+^ T cell population.

Regarding the correlations between RORC expression and MHC molecules (which are closely associated with cancer occurrence and development (Cornel et al., 2020)), RORC expression exhibited the strongest positive correlation with HLA-F in ACC, and the strongest negative correlation with B2M in UVM. Although an association between HLA-F and ACC has rarely been reported, the differential HLA-F expression in STAD, BRCA, and COAD (relative to normal tissue) has been confirmed (Ishigami et al., 2015; Huang et al., 2020; Wuerfel et al., 2020). B2M is a critical molecule in tumorigenesis and immune monitoring and is considered a latent CIT target (Wang et al., 2021). On the basis of the correlations between RORC expression and the immunomodulators mentioned above, we propose that RORC may be a potential CIT target for treating certain cancers.

PD-L1 expression is considered an effective biomarker that can predict the clinical efficacy of PD-1/PD-L1 inhibitors (Davis and Patel, 2019), and MSI and TMB are also effective CIT biomarkers (Le et al., 2015; Romero, 2019). Therefore, we further studied the correlations of RORC expression with PD-L1, TMB, and MSI. RORC was negatively associated with PD-L1 and TMB in BRCA, THCA, and THYM, and negatively correlated with TMB and MSI in LUSC. Notably, RORC was correlated (mostly negatively) with PD-L1 in most cancers. Lytle et al. (Lytle et al., 2019) found that inhibiting RORC expression hinders the progression of PAAD, which they believe may represent a new treatment strategy for PAAD. Interestingly, our results showed that in PAAD, RORC expression was negatively correlated with PD-L1, suggesting that high RORC expression may be a reason for the insensitivity to anti-PD-L1 treatment in PAAD. These findings suggest that low RORC expression may improve CIT responses in certain cancers. There was a non-significant tendency for the low RORC expression subgroups to exhibit a better CIT response than the high RORC expression subgroups in the three independent CIT cohorts. Because only three relevant cohorts were explored in this study, it is difficult to comprehensively elucidate the CIT response in relation to RORC expression levels. More CIT cohorts should be studied in the future.

In addition, we must acknowledge that our study has several further limitations. In particular, the data on RORC gene expression in tumor and normal tissues, as well as the data showing the association between RORC expression and cancer prognosis, were obtained retrospectively from public databases. Although our study has obtained useful findings, validation involving animal experiments and prospective multicenter, large-sample studies is required.

## Conclusions

This study comprehensively demonstrates the important role that RORC plays in immunity in many cancers. These findings provide preliminary evidence for the application of RORC in CIT.

## Data Availability

The original contributions presented in the study are included in the article/Supplementary Material, further inquiries can be directed to the corresponding authors.
