# Supplementary figures and images for: A comprehensive pancancer analysis reveals the potential value of RAR-related orphan receptor C (RORC) for cancer immunotherapy
Source: Front Genet. 2022 Sep 15;13:969476. doi: 10.3389/fgene.2022.969476 (PMC9520743; doi:10.3389/fgene.2022.969476)

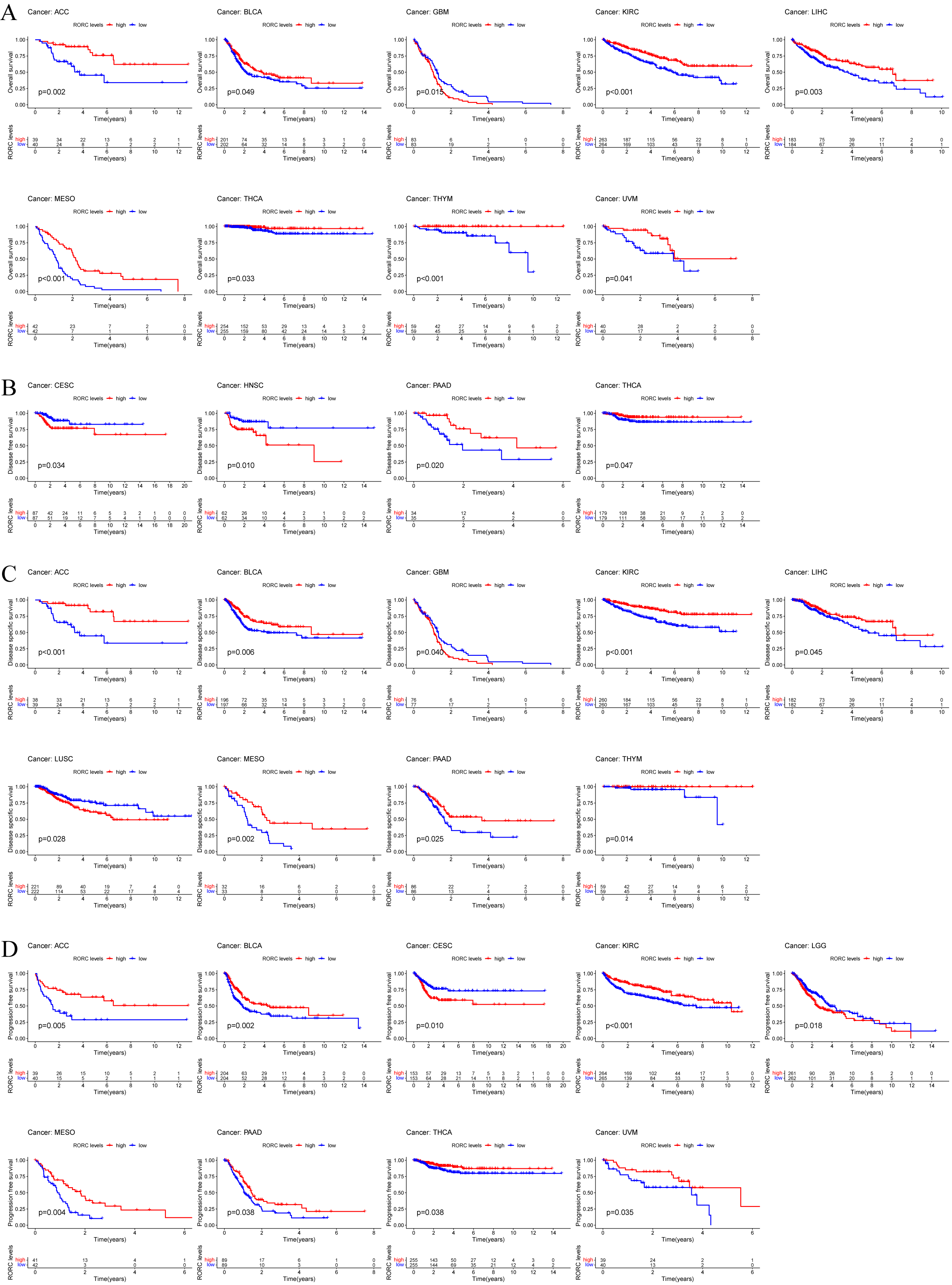

Supplement: Supplementary file 1 [file Image1.TIF]
